# Impact of prenatal and childhood adversity effects around World War II on multimorbidity: results from the KORA-Age study

**DOI:** 10.1186/s12877-022-02793-2

**Published:** 2022-02-11

**Authors:** Ava Arshadipour, Barbara Thorand, Birgit Linkohr, Susanne Rospleszcz, Karl-Heinz Ladwig, Margit Heier, Annette Peters

**Affiliations:** 1 Institute of Epidemiology, Helmholtz Zentrum München, German Research Center for Environmental Health, Neuherberg, Ingolstädter Landstraße 1, 85764 München, Germany; 2 Institute for Medical Information Processing Biometry and Epidemiology (IBE), Ludwig-Maximilians-Universität München, München, Germany; 3 German Center for Diabetes Research (DZD), München-Neuherberg, Germany; 4 Department for Psychosomatic Medicine and Psychotherapy, Klinikum Rechts Der Isar, Technical University of München, München, Germany; 5 KORA Study Centre, University Hospital of Augsburg, Augsburg, Germany; 6 German Center for Cardiovascular Disease Research (DZHK), München Heart Alliance, München, Germany

**Keywords:** Chronic disease, Multimorbidity, Geriatrics, Logistic regression, Machine learning, Agglomerative hierarchical clustering

## Abstract

**Background:**

While risk factors for age-related diseases may increase multimorbidity (MM), early life deprivation may also accelerate the development of chronic diseases and MM.

**Methods:**

This study explores the prevalence and pattern of MM in 65–71 year-old individuals born before, during, and after World War II in Southern Germany based on two large cross-sectional KORA (Cooperative Health Research in the Region of Augsburg) -Age studies in 2008/9 and 2016. MM was defined as having at least two chronic diseases, and birth periods were classified into five phases: pre-war, early war, late war, famine, and after the famine period. Logistic regression models were used to analyze the effect of the birth phases on MM with adjustment for sociodemographic and lifestyle risk factors. Furthermore, we used agglomerative hierarchical clustering to investigate the co-occurrence of diseases.

**Results:**

Participants born during the late war phase had the highest prevalence of MM (62.2%) and single chronic diseases compared to participants born during the other phases. Being born in the late war phase was significantly associated with a higher odds of MM (OR = 1.83, 95% CI: 1.15–2.91) after adjustment for sociodemographic and lifestyle factors. In women, the prevalence of joint, gastrointestinal, eye diseases, and anxiety was higher, while heart disease, stroke, and diabetes were more common in men. Moreover, three main chronic disease clusters responsible for the observed associations were identified as: joint and psychosomatic, cardiometabolic and, other internal organ diseases.

**Conclusions:**

Our findings imply that adverse early-life exposure may increase the risk of MM in adults aged 65–71 years. Moreover, identified disease clusters are not coincidental and require more investigation.

**Supplementary Information:**

The online version contains supplementary material available at 10.1186/s12877-022-02793-2.

## Introduction

Parallel to the worldwide increase in life expectancy in the last decades, the prevalence of age-related chronic diseases has also risen. Consequently, multimorbidity (MM), defined as the presence of at least two chronic diseases has become increasingly prevalent especially among older adults. MM is a major concern in the health care system since it can result in reduced quality of life, increased mortality, disability, and higher health care costs [1].

Based on a systematic literature review of 41 articles from different countries, the prevalence of MM ranges from 55 to 98% in those aged ≥ 65 years [2]. In Germany, based on the cross-sectional national telephone health interview survey "German Health Update" (GEDA 2012–2013), the MM prevalence ranged from 61.7% (95% CI: 59.3 -64.1) for 60 to 69 year-old to 72.9% (95% CI: 70.4–75.2) for 70 to 79 year-old individuals [3]. Others reported a 62% MM prevalence for those aged ≥ 65 years in the German population [4]. In Augsburg, MM prevalence was 58.6% for individuals aged 65–94 years based on the KORA-Age 1 data in 2008/9 [5]. Various studies have assessed the association of MM with sociodemographic and lifestyle factors, which indicated MM has been mainly associated with age, sex, educational level, income, physical activity, smoking, and alcohol consumption [6, 7].

The potential clustering of chronic diseases is in particular relevant for prevention, diagnosis, and treatment. Various studies have explored different approaches to determine the co-occurrence of chronic diseases, such as analyzing the prevalence of specific disease combinations, network analysis, factor analysis, and multiple correspondence analysis [8]. Kirchberger et al. [5] used factor analysis based on the Tetrachoric correlation matrix for 4127 persons. They identified four main comorbidity patterns: metabolic/cardiovascular diseases, liver/lung/joint/eye diseases, anxiety/depression/neurological diseases, and cancer/gastrointestinal problems. To specify the chronic diseases clustering, only a few studies to date used clustering as an unsupervised machine learning algorithm, which has some advantages: No initial assumption on data distribution, the number of clusters or cluster structure is needed in most algorithms and the results are very informative by using dendrograms for visualization [8].

Childhood adversity and malnutrition have been identified in studies as a possible source of chronic disease risk and then MM in later life [9]. In addition, socioeconomic hardship and traumatic events such as abuse and neglect during early life stages can contribute to chronic diseases progression [10, 11]. Regarding the early life experience, it is also worth noting that there were widespread food shortages during World War II, especially in the Soviet Union, India, China, Java, Vietnam, Greece, Austria, and The Netherlands. However, in Germany, food shortages mainly occurred at the end of World War II due to the destruction of agricultural land, livestock, machinery, and labor shortages. In Germany, adults aged 65–71 years who born or were in their crucial developmental age during the famine period and the early reconstruction after World War II had increased risk of health deficit accumulation including chronic diseases, disability and frailty. The average energy consumption per person in Germany decreased from about 2500 cal in 1944 to 1050–1250 cal in 1946, and after June 1948, it increased to 1800 cal again due to the currency reform [12]. Since aged adults in our sample born in Germany from 1937 to 1950 were exposed to this crucial period in their early phases of life, it offers the opportunity to study how the adversity of Word War II affected the prevalence of MM and chronic diseases later in life.

Therefore, we aimed to determine the effect of adverse developmental age situations on MM development and single chronic diseases in older individuals aged 65–71 years. In addition, we explored the pattern of chronic diseases to specify the clusters of these chronic diseases for this age group.

## Methods

### Data collection and study sample

**Fig. 1:**
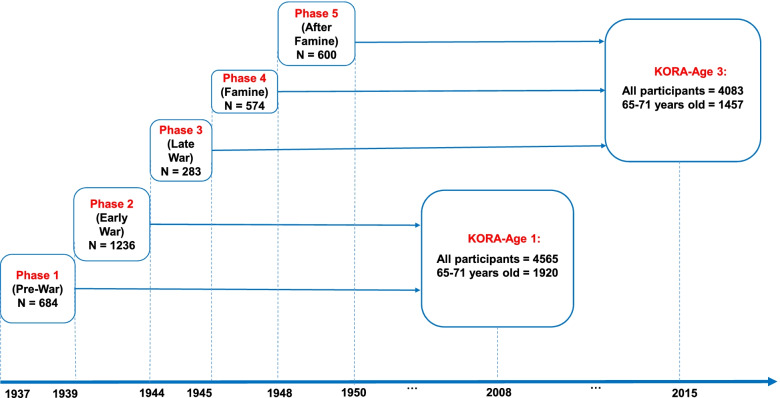
Participants of the KORA-Age 1 and KORA-Age 3. Combined study population (*N* = 3,377) and birth phases. 2008 and 2015 referred to the years for the age calculation in two studies. Phases were defined based on participants critical developmental age (prenatal gestation or the first two years of life) and the World War II situation in Germany

Data originated from two population-based cross-sectional KORA-Age study arms: KORA-Age 1 conducted from 01.12.2008 to 06.11.2009 and KORA-Age 3 conducted from 01.02.2016 to 07.10.2016, which are follow-ups of four independent cross-sectional studies (S1 1984/5, S2 1989/90, S3 1994/5, and S4 1999- 2001). Both studies focused on the health of participants aged 65 and older in Augsburg and the two adjacent regions in Southern Germany based on questionnaires and telephone interviews. The KORA-Age study is described in detail elsewhere [13]. In short, 4,565 out of 5991 eligible individuals (response rate: 76.2%) participated in the KORA-Age 1 survey, and 1920 were between 65 and 71 years old on 31.12.2008 (born between 1937–1943). The KORA-Age 3 survey consisted of 4,083 out of 6051 eligible individuals (response rate: 67.5%), of whom 1444 participants were in the same age range on 31.12.2015 (born between 1944–1950). The present analysis combines these two similar KORA-Age study arms, which were completely independent because of the different birth years of the participants (Fig. 1).

### Exposure: birth phases

Since malnutrition during pregnancy and early childhood can have a negative impact on metabolism, growth and development of chronic diseases later in life [9], the exposure variable was defined based on the participants’ crucial developmental age during prenatal gestation or the first two years of life. Based on the World War II situation and the famine period which occurred afterwards roughly until June 1948 in Germany, individuals were divided into five independent birth phases: pre-war (January 1937-August 1939), early war (September 1939-December 1943), late war (January 1944-April 1945), famine period after the war (May 1945- Jun 1948) and after famine period and reconstruction (July 1948-Dec 1950) [12].

### Outcome: multimorbidity

The primary outcome was MM, defined as the presence of two or more concomitant chronic diseases in individuals [5]. We considered fourteen major chronic diseases, including hypertension, eye disease, heart diseases, diabetes, joint disease, lung disease, gastrointestinal disease, stroke, cancer, kidney diseases, liver diseases, neurological diseases, depression, and anxiety. All disease variables were defined as life-time diagnoses except for cancer diagnosed within the last three years. Hypertension, diabetes, cancer, stroke, heart diseases (myocardial infarction and coronary artery disease) were assessed based on the questionnaire. All other diseases were identified in a telephone interview based on the Charlson Comorbidity Index [14]. Participants were asked whether they suffer from kidney, liver, lung diseases (e.g., asthma, chronic bronchitis, and emphysema), inflammatory joint problems (e.g., arthritis or rheumatism), gastrointestinal diseases (e.g., colitis, cholecystic, gastric, or ulcer), heart diseases (e.g., congestive heart failure, coronary heart failure, or angina), eye problem (e.g., cataract, retinitis pigmentosa, glaucoma, macular degeneration, diabetic retinopathy). Neurological diseases were evaluated based on diseases like epilepsy, Parkinson's, or sclerosis using telephone interviews. The Geriatric Depression Scale [15] and Generalized Anxiety Disorder Scale-7 [16] were used to diagnose depression and anxiety. Individuals with scores ≥ 10 were defined as suffering from depression or anxiety.

### Explanatory variables

We considered age, sex, education level, alcohol consumption, physical activity, body mass index (BMI), smoking behavior, and cognitive status as covariates. Education levels were based on the duration of education and vocational training and categorized into three groups: low (9 years or less), middle (10 or 11 years), and high (12 years or more). Body mass index (BMI), defined by the World Health Organization (WHO), was used. Participants were categorized as underweight or normal weight (BMI < 24.99 kg/m^2^), overweight (25 ≤ BMI ≤ 29.99), obese class I (30 ≤ BMI < 34.99), and obese class II or III (35 ≤ BMI) [17].

Leisure time physical activity was measured from two separate questions about leisure time sports activity in winter and summer, including cycling. Possible answers were (1) > 2 h, (2) 1–2 h, (3) < 1 h and (4) none. Participants, who had a total score less than 5, obtained by summing the numbers (1)–(4) relating to activities in winter and summer, were classified to be “physically active” [18].

Alcohol consumption was based on self-reported alcohol intake with the following five groups: ‘‘almost every day’’, ‘‘several times a week’’, ‘‘about once a week’’, ‘‘less than once a week’’, and “never or seldom” [19]. For our analysis, we categorized the first two groups as “daily use” and the last two as “never or rare use.” Based on self-reported information, there are three categories for smoking status: never smokers, former smokers, and active smokers. The cognitive status is identified as dementia, mildly impaired cognitive status, and normal status based on TICS-M (Telephone Interview for Cognitive Status) score, a standard instrument for assessing cognitive impairment [20].

### Statistical analysis

The frequency and prevalence of baseline characteristics were stratified by sex and birth phases, and the Chi-squared test was computed to check the differences. Overall, the stratified prevalence of MM and single diseases was calculated and tested by the Chi-squared test. Then Post-hoc tests with Bonferroni adjustment were performed for multiple comparisons. Covariates multi-collinearity was assessed using the variance inflation factors (VIF). Associations between the birth phases and MM were estimated by odds ratios (OR) in logistic regression models with different adjustment steps for risk factors. We used a standardized age variable (calculated as age minus mean age divided by the standard deviation of age) in our models because of different age distributions in the different birth phases. The modeling process started with the standardized age variable as covariate only (model 1), then birth period only (model 2), standardized age (rescaled with mean and standard deviation) and birth period together (model 3), then sex, education, alcohol use, physical activity, BMI, smoking behavior and cognitive status were added to the final model (models 4). The interaction effect of sex and birth phase variable was also checked in the final model. Agglomerative hierarchical clustering approach as an unsupervised machine learning technique was carried out to identify disease clusters so that diseases in one cluster are more similar than diseases in other clusters. This bottom-up algorithm begins with each disease as an individual cluster and merges the similar clusters until remaining only one cluster based on the proximity distance matrix. The average linkage method as proximity distance and Yule Q coefficient as similarity measurement for the binary disease variables were considered. The final cluster selection was created based on the threshold (cutoff height), corresponding to subject information, prior research, and clinical significance.

Since there is a big difference between the prevalence of hypertension and other chronic diseases, regression models and cluster analysis were performed without hypertension as a sensitivity analysis. Furthermore, the Ward and Single linkage methods, as other possible determinants for the pairwise distance between the set of observations were used to determine the robustness of agglomerative hierarchical clustering [8]. As our analysis was based on the combination of two very similar KORA-Age studies conducted in 2008/9 and 2016, we examined the effect of study period in another sensitivity analysis:. To see if the differences across phases are indeed due to differences in phases rather than a study effect, we used the generalized linear mixed models (GLMM) and created two GLMMs, one with a phase variable as a random effect only and one with a phase variable as a random effect nested in the study variable.

## Results

### Study population characteristics

The final sample consisted of 3377 participants aged 65 to 71 years (Fig. 1). From this population, 684 persons (52.2% female) were born during the pre-war, 1236 persons (50.8% female) in the early war, 283 persons (51.9% female) in the late war, 574 persons (53.5% female) in the famine period, and 600 persons (53.7% female) after the famine phase.

The overall baseline characteristics of the participants and their stratification by each phase are displayed in Table 1. Men were more likely to have a high educational level (45.0% versus 19.7%, *p* < 0.001), drink alcohol daily (65.1% versus 29.8%, *p* < 0.001), have pre-obesity (52.1% versus 38.9%, p < 0.001) or obesity class I (20.4% versus 17.0%, *p* < 0.001), be a former smoker (57.5% versus 32.2%, *p* < 0.001) and have a slightly impaired cognitive status (8.7% versus 4.8%, *p* < 0.001) compared with women.

**Table 1:** Descriptive statistics percentages stratified by sex and birth phases for the whole participants (*n* = 3,377) from KORA-Age 1 and KORA-Age 3

Characteristic		Total (*N* = 3,377)	Sex			Birth phases					
			Male (*N* = 1,616)	Female (*N* = 1,761)	*P*-value	Pre-war (*N* = 684)	Early war (*N* = 1,236)	Late war (*N* = 283)	Famine (*N* = 574)	After famine (*N* = 600)	*P*-value
Sex	Male	47.8				47.8	49.2	48.1	46.5	46.3	0.76
	Female	52.2				52.2	50.8	51.9	53.5	53.7	
Age mean (SD)	MM = yes	68.1 (1.9)	68.1 (2.0)	68.2 (1.9)	0.325	70.1 (0.8)	66.9 (1.3)	70.7 (0.42)	68.5 (0.9)	65.9 (0.7)	0.750
	MM = no	67.7 (1.9)	67.7 (1.9)	67.7 (1.9)	0.364	70.1 (0.7)	66.8 (1.2)	70.7 (0.4)	68.5 (0.9)	65.8 (0.7)	0.734
Education	Low	10.2	3.2	16.6	< 0.001	16.5	11.6	5.6	5.4	6.7	< 0.001
	Middle	58.0	51.8	63.7		57.7	58.5	56.9	59.7	56.2	
	High	31.7	45.0	19.7		25.7	29.8	37.1	34.8	37.0	
Alcohol consumption	Never or rare use	38.5	20.9	53.8	< 0.001	38.6	37.1	38.2	40.9	36.7	0.038
	Once a week	15.1	13.9	16.3		12.4	15.1	20.5	13.7	17.2	
	Daily use	46.7	65.1	29.8		49.0	47.8	41.3	44.9	45.8	
Physical Activity	Active	66.8	69.2	64.6	0.005	64.5	66.6	63.6	67.1	71.3	0.072
	Inactive	33.2	30.8	35.4		35.5	33.3	36.4	32.7	28.7	
BMI	Underweight or normal weight	29.1	22.4	35.4	< 0.001	26.9	27.5	32.9	29.9	32.5	< 0.001
	Pre obese	45.2	52.1	38.9		48.2	47.1	46.2	41.0	41.5	
	Obese class I	18.6	20.4	17.0		19.9	19.6	12.0	19.3	17.5	
	Obesity class II or III	6.2	4.4	7.9		4.2	5.3	6.7	8.4	8.0	
Smoking behavior	Never	45.3	31.6	58	< 0.001	55.9	45.4	43.8	39.7	39.0	< 0.001
	Active smoker	10.3	10.9	9.8		7.3	10.1	10.6	9.4	15.0	
	Former smoker	44.3	57.5	32.2		36.8	44.3	45.6	50.9	46.0	
Cognitive status	Good	89.5	86.5	92.3	< 0.001	84.8	90.8	86.2	92.0	91.5	< 0.001
	Mildly impaired	6.6	8.7	4.8		10.0	5.6	8.1	5.2	5.7	
	Impaired	2.1	2.8	1.5		3.0	2.3	2.8	1.4	1.2	
Baseline Survey	S1	26.0	25.3	26.7	0.685	27.6	26.8	24.0	24.8	24.7	0.17
	S2	23.5	23.4	23.6		21.1	25.0	25.1	23.2	22.5	
	S3	25.8	25.7	25.8		28.9	24.9	26.1	24.0	25.5	
	S4	24.7	25.6	24.0		22.4	23.2	24.7	28.0	27.3	

Values represent % unless otherwise indicated. *P*-value from Chi-square test for categorical variables, from t-Test for comparing the mean of age variable and from ANOVA test for comparing the mean of multiple phases. Phases were defined based on participant’s critical developmental age (prenatal gestation or the first two years of life) and the World War II situation in Germany

Educational levels increased over time with the lowest level of high education for people born before the war (26% high education) and higher levels later (37% high education in after famine). Individuals born during and after the famine had a lower percentage (41.0% and 41.5%, *p* < 0.001) of pre-obesity compared to the other phases. Participants born after the famine were more likely (15.0%) to be active smokers than people born before. Individuals born during the late war phase had a higher percentage of mildly impaired (8.1%) and impaired cognitive (2.8%) status compared with other phases (Table 1).

### Prevalence of MM and single chronic diseases

MM prevalence was 49.4% in the total sample. There were no considerable differences in the prevalence of MM among men (48.8%) and women (49.9%) overall. MM prevalence was highest (62.2%) for individuals born during late war phase compared with the other phases (Table 2). In post hoc multiple comparisons, significant differences in MM prevalence were observed between late war and early war phase and between late war and after famine phase. There was no difference in MM between men and women (Fig. S1). Likewise, there were the same pattern in males and females and multiple comorbidities (Table S1). The prevalence of the chronic diseases stratified by sex and phases was presented in Table 2. Overall, hypertension (56.7%), eye diseases (25.6%), and heart diseases (18.9%) were the most prevalent diseases in both men and women. Although, women had significantly higher prevalence of joint (13.1% vs. 7.4%, *p* < 0.001), gastrointestinal (9.1% vs. 6.7%, *p* = 0.01), eye diseases (28.5% vs. 22.5%, *p* < 0.001) and anxiety (7.8% vs. 3.4%, *p* < 0.001), men had more heart diseases (23.0% vs. 15.1%, *p* < 0.001), stroke (6.8% vs. 3.1%, *p* < 0.001) and diabetes (17.1% vs. 12.9%, *p* < 0.001). Furthermore, there was a significant difference in the prevalence of some single diseases according to the birth phases. For most diseases, the prevalence was highest among individuals born in late war (Table 2).

**Table 2:** Number and percentage of single diseases and multimorbidity stratified by sex and birth phases for the whole participants (*n* = 3,377) from KORA-Age 1 and KORA-Age 3

Disease	Total (3,377)	Sex			Birth phases					
		Male (1,616)	Female (1,761)	P-value	Pre-war (684)	Early war (1,236)	Late war (283)	Famine (574)	After famine (600)	P-value
Multimorbidity (MM)	1667 (49.4%)	788 (48.8%)	879 (49.9%)	0.503	370 (54.1%)	561 (45.4%)	176 (62.2%)	310 (54.0%)	250 (41.7%)	< 0.001
Lung	343 (10.2%)	157 (9.7%)	186 (10.6%)	0.415	61 (8.9%)	109 (8.8%)	35 (12.4%)	71 (12.4%)	67 (11.2%)	0.065
Joint diseases( Arthritis. Rheumatic)	349 (10.3%)	119 (7.4%)	230 (13.1%)	< 0.001	74 (10.8%)	138 (11.2%)	31 (11.0%)	52 (9.1%)	54 (9.0%)	< 0.001
Cancer	145 (4.3%)	74 (4.6%)	71 (4.0%)	0.433	25 (3.7%)	55 (4.4%)	13 (4.6%)	27 (4.7%)	25 (4.2%)	0.898
Gastrointestinal	270 (8%)	109 (6.7%)	161 (9.1%)	0.0101	61 (8.9%)	97 (7.8%)	21 (7.4%)	49 (8.5%)	42 (7.0%)	0.735
Heart diseases	637 (18.9%)	371 (23%)	266 (15.1%)	< 0.001	149 (21.8%)	216 (17.5%)	65 (23.0%)	121 (21.1%)	86 (14.3%)	< 0.001
Stroke	165 (4.9%)	110 (6.8%)	55 (3.1%)	< 0.001	34 (5.0%)	52 (4.2%)	27 (9.5%)	32 (5.6%)	20 (3.3%)	< 0.001
Kidney	117 (3.5%)	62 (3.8%)	55 (3.1%)	0.257	30 (4.4%)	34 (2.8%)	18 (6.4%)	24 (4.2%)	11 (1.8%)	0.002
Liver	82 (2.4%)	43 (2.7%)	39 (2.2%)	0.400	15 (2.2%)	29 (2.3%)	9 (3.2%)	15 (2.6%)	14 (2.3%)	0.913
Diabetes mellitus	504 (14.9%)	276 (17.1%)	228 (12.9%)	< 0.001	118 (17.3%)	165 (13.3%)	48 (17.0%)	95 (16.6%)	78 (13.0%)	0.056
Neurological disease	103 (3.1%)	51 (3.2%)	52 (3.0%)	0.731	22 (3.2%)	31 (2.5%)	10 (3.5%)	20 (3.5%)	20 (3.3%)	0.727
Hypertension	1914 (56.7%)	910 (56.3%)	1004 (57.0%)	0.626	396 (57.9%)	673 (54.4%)	179 (63.3%)	344 (59.9%)	322 (53.7%)	0.013
Eye disease	866 (25.6%)	364 (22.5%)	502 (28.5%)	< 0.001	202 (29.5%)	285 (23.1%)	103 (36.4%)	164 (28.6%)	112 (18.7%)	< 0.001
Anxiety	192 (5.7%)	55 (3.4%)	137 (7.8%)	< 0.001	55 (8.0%)	87 (7.0%)	9 (3.2%)	24 (4.2%)	17 (2.8%)	< 0.001
Depression	44 (1.3%)	16 (1.0%)	28 (1.6%)	0.131	12 (1.8%)	15 (1.2%)	3 (1.1%)	9 (1.6%)	5 (0.8%)	0.616

*P*-value from Chi-square test for categorical variables. Phases were defined based on participant’s critical developmental age (prenatal gestation or the first two years of life) and the World War II situation in Germany

### Association between birth phase and MM

In the age-adjusted logistic regression model, the odds ratio of MM in late war phase (OR = 1.69, 95% CI: 1.08–2.64) and famine phase (OR = 1.39, 95% CI: 1.04–1.87) were significantly higher compared to the reference after famine phase. After adjusting for other covariates (model 4), participants born in late war had a higher odds of MM compared with the individuals born in after famine phase (OR = 1.83, 95% CI: 1.15–2.91) (Table 3). We did not observe any significant interaction between sex and birth phase on the odds of MM (Fig. S1).

**Table 3:** Odds ratios and 95% confidence intervals for multimorbidity based on hierarchical logistic regression for the whole participants (*n* = 3,377) from KORA-Age 1 and KORA-Age 3

Characteristics		model 1	model 2	model 3	model 5
Standardized age		1.12 (1.08–1.16)		1.12 (0.98–1.29)	1.11 (0.96–1.27)
Birth phases (ref: After famine)	Pre-war		1.64 (1.32–2.05)	1.26 (0.88–1.82)	1.35 (0.92–1.98)
	Early war		1.16 (0.95–1.41)	1.09 (0.88–1.34)	1.08 (0.87–1.34)
	Late war		2.3 (1.72–3.07)	1.69 (1.08–2.64)	1.83 (1.15–2.91)
	Famine		1.64 (1.30–2.07)	1.39 (1.04–1.87)	1.35 (0.99–1.84)
Female (ref: male)					0.98 (0.83–1.16)
Education (ref: low)	Middle				0.82 (0.63–1.05)
	High				0.72 (0.55–0.95)
Alcohol consumption (ref: never or rare use)	Once a week				0.99 (0.81–1.20)
	Daily use				0.76 (0.64–0.91)
Physical Activity (active)	Inactive				1.47 (1.26–1.72)
BMI (ref: underweight or normal)	Overweight				1.32 (1.11–1.57)
	Obesity class I				1.92 (1.55–2.38)
	Obesity class II or III				3.09 (2.21–4.33)
Smoking behavior (ref: never smoker)	Active smoker				1.34 (1.05–1.72)
	Ex-smoker				1.36 (1.16–1.59)
Cognitive status (ref: good)	Mildly impaired				1.43 (1.07–1.92)
	Impaired				1.32 (0.81–2.18)
AIC		4638.4	4639.0	4637.8	4373.6

Phases were defined based on participant’s critical developmental age (prenatal gestation or the first two years of life) and the World War II situation in Germany

### The pattern of comorbidity

**Fig. 2:**
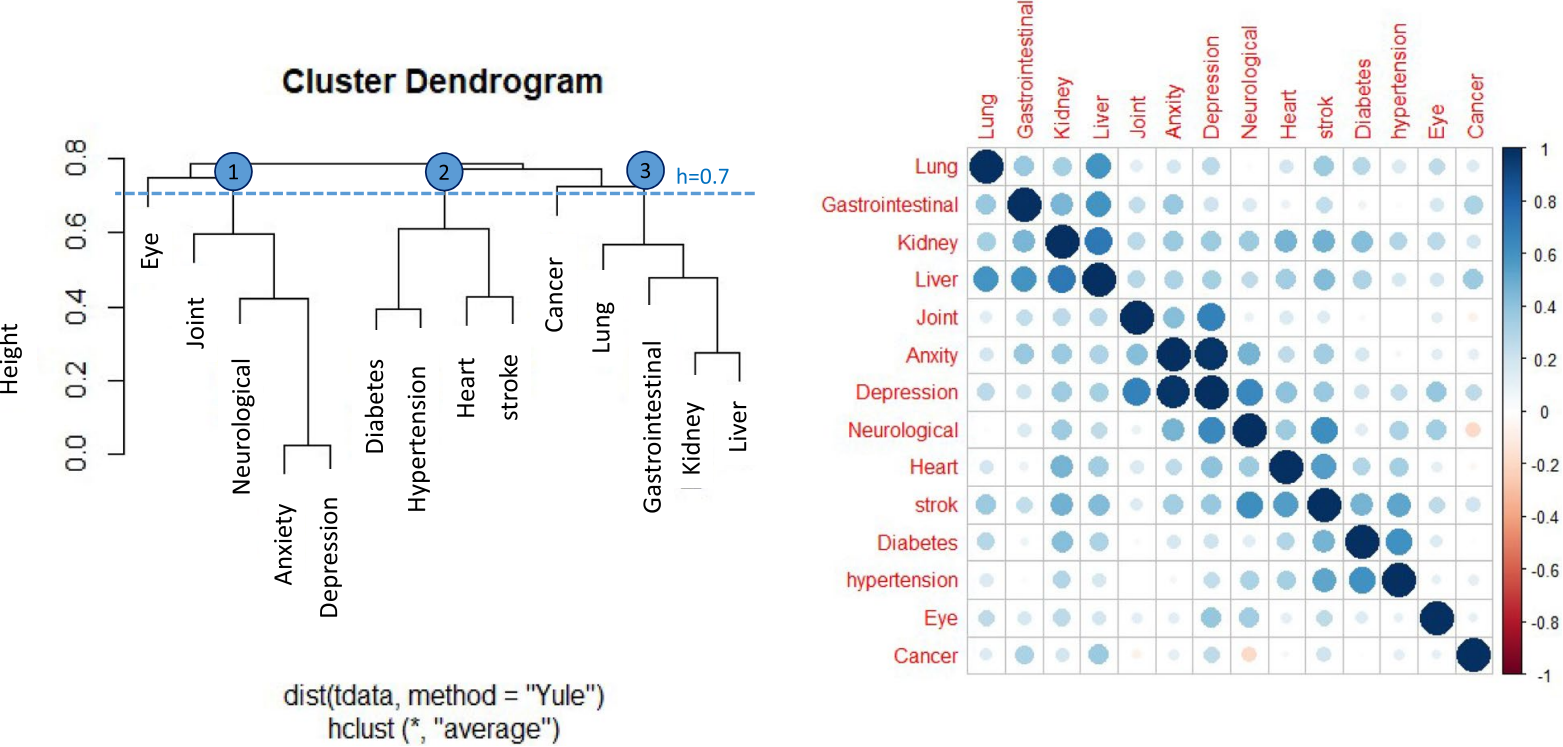
*Left:* Dendrogram based on the hierarchical clustering of chronic diseases using the average linkage method and Yule-Q coefficient. The threshold (dotted line, h=0.7) was used to specify three main clusters for all participants; *Right:* Correlation matrix of chronic diseases based on Yule coefficient. Three red squared lines shows three main clusters on the matrix

Three main clusters of diseases were specified based on the agglomerative hierarchical clustering. The first cluster composed of joint and psychosomatic disorders consisted of anxiety, depression, joint and neurological diseases. The second cluster of cardio-metabolic diseases comprised of diabetes, hypertension, stroke, and heart diseases. The last one was the other internal organ diseases cluster, including the lung, gastrointestinal, kidney, and liver diseases (Fig. 2).

The number and percent of multimorbid individuals for each cluster were calculated by dividing the number of individuals who had at least two of these diseases in the cluster by the total number of multimorbid patients. In total, 1002 of the 1667 participants with MM could be assigned to at least one cluster. A high percentage (49.1%) of multimorbid participants were assigned to the cardiometabolic cluster. The other internal organ cluster had a similar prevalence in terms of sex and different phases of the birth period; however, the joint and psychosomatic diseases cluster had a more significant prevalence in women (6.4%) and pre-war phase (7.0%) and early war phase (6.1%) of birth years. Moreover, men (57.9%) and participants born in pre-war (52.7%) and late war (52.8%) had a significantly higher prevalence in the cardiometabolic disease cluster (Table 4).

**Table 4:** Number and percent of individuals with multimorbitiy (i.e. at least two diseases) in each cluster

Cluster	Total (1,667)	Sex			Birth phases					
		Male (788)	Female (879)	*P*-value	Pre-war (370)	Early war (561)	Late war (176)	Famine (310)	After famine (250)	*P*-value
Joint. Neuro. Anxiety. Depression	78 (4.7%)	22 (2.8%)	56 (6.4%)	< 0.001	26 (7%)	34 (6.1%)	2 (1.1%)	9 (2.9%)	7 (2.8%)	0.002
Heart. Stroke. Diabetes. Hypertension	818 (49.1%)	456 (57.9%)	362 (41.2%)	< 0.001	195 (52.7%)	258 (46%)	93 (52.8%)	156 (50.3%)	116 (46.4%)	< 0.001
Lung. Gastro. Kidney. Liver	106 (6.4%)	44 (5.6%)	62 (7.1%)	0.218	20 (5.4%)	31 (5.5%)	17 (9.7%)	19 (6.1%)	19 (7.6%)	0.051

*P*-value from Chi-square test for categorical variables. Phases were defined based on participant’s critical developmental age (prenatal gestation or the first two years of life) and the World War II situation in Germany

### Sensitivity analysis

We repeated the analysis without hypertension for MM and the findings were close to the previous model analysis including hypertension overall. Late war Phase still had the highest MM odds ratio (OR = 2.14, 95% CI: 1.29–3.52). Moreover, pre-war phase (OR = 1.64, 95% CI: 1.07–2.49) and famine phase (OR = 1.52, 95% CI: 1.08–2.14) had a bit higher odds than hypertension consideration, and they were significant (Table S2). When results from GLMM with a phase variable as random effect were compared to results from a logistic regression model with a phase variable as fixed effect, there was no significant difference in the covariates effect estimate. Moreover, considering a phase variable nested in the study did not significantly improve the model compared to considering phase as random effect only (Table S3).

Hierarchical clustering was also performed without hypertension, and the three major clusters remained as before (Fig. S5). The dendrograms of chronic diseases association based on the Single and Ward linkage approaches were also very close to the average method. The number of main clusters and included diseases also remained stable (Fig. S6, S7).

## Discussion

### Multimorbidity

In our study, the overall prevalence of MM was 49.4% for adults aged 65–71 years-old. The comparison of the prevalence of MM between studies is hampered by differences in the examined age groups, including diseases and study areas, even in Germany [3]. While there was no difference between men and women in the MM prevalence in the present study like in other studies [4, 7], others observed a higher prevalence in women [21]. Although men had a higher prevalence of diabetes, heart disease, and stroke relative to women, the prevalence of joint, gastrointestinal, and eye diseases and anxiety was more remarkable for women in the present study.

### Multimorbidity according to birth phases

The prevalence of MM and every single chronic disease was higher in late war phase. Since it is well known that MM and chronic diseases increase with increasing age [2], differences in the unadjusted prevalence could be biased by differences in the age distribution between birth phases. Therefore, we also assessed the impact of the birth phase on MM in logistic regression models adjusted for age and other sociodemographic and lifestyle factors. We found that the OR of MM was significantly higher in late war compared with after famine, even after adjusting for these potential confounders. This effect could be explained by the unfavorable living conditions which were observed in the late war phase in South Germany. Large parts of the city of Augsburg were devastated during the most extensive bombing raid at the end of February 1944 [22]. Participants born during the last years of war were thus exposed to food crises and famine in Germany in 1945 during early life stages [12]. In this context, it has previously been shown that maternal and early-life malnutrition can negatively affect adults mental and physical health [23]. Individuals with low birthweight could have negative outcomes later in life. They might have poorer cognitive performance, higher blood pressure, decreased glucose tolerance, poorer functionality in lung, kidney and immune system, more diseases like coronary heart disease, chronic lung and kidney disease, diabetes and higher cardiovascular and all-cause mortality [9].

Moreover, an increased chronic health disease incidence was identified for aged individuals (born 1922–1960 in former West Germany) exposed to war during their utero and childhood [24]. These findings are important as they indicate that besides well-established risk factors in adult life, the birth phases and the living conditions during the World War II still are of concern today. Therefore, health service measures as well as individual treatment efforts may specifically need to pay attention to both men and women born between 1944 to 1945.

### Clusters of chronic diseases

We identified three major clusters with the clustering approach to recognize individuals with similar MM diseases. The other internal organ cluster included illnesses involving main body organs, such as the stomach and intestines, kidneys, liver, and lung, which indicated the same co-occurrence in men and women and individuals born in different phases. Association among the lung, liver, and gastrointestinal diseases is consistent with Rodriguez-Roisin et al. [25] in adult patients based on the possible shared mechanisms. This coexistence might increase vascular diseases, such as portopulmonary hypertension and hepatopulmonary syndrome, and other chronic respiratory diseases coexisting with chronic hepatic diseases. Still, more research is warranted to corroborate our understanding of the underlying mechanisms for this cluster.

We verified the association between joint diseases and psychosomatic disorders within the second cluster, more prevalent in women. Previous studies have established links between rheumatoid arthritis, mood disturbances, and neurological diseases [26, 27]. Lee et al. [26] observed the coexistence of Parkinson's disease and rheumatics. Lwin et al. [27] also observed that depression was twice as prevalent in patients with rheumatoid arthritis as in the general population.

The cardiometabolic cluster had the highest proportion of co-occurrence of cardiovascular and metabolic diseases, typically found in aged people, and it was more prevalent in men. This relationship also has been widely illustrated in prior populations [5, 28]. Sowers et al. [28] showed that hypertension prevalence was twice as high in people with diabetes than those without diabetes. Also, individuals with hypertension experienced diabetes more frequently than persons with normal blood pressure. They also reported that hypertension could be responsible for up to 75% of CVD in diabetes.

### Strengths and weaknesses

One of our analysis strengths is that it was based on two large data sets from the KORA cohort study [13] with individuals born around World War II. This enabled us to analyze comorbidity and MM in different birth phases. The information also came from the specific age range of individuals from two KORA-Age studies that provided uniformity in data, which is essential for exploring disease patterns. This huge database also contained information about demographic, sociodemographic, physical, and mental health factors, which helped adjust a wide range of factors associated with MM. Another strength is that both cross-sectional KORA-Age studies used the same instruments in the interview; thus the data is less likely to be biased [29]. The clustering method helped discover disease comorbidity clusters that define specific risk domains and assign individuals to subgroups with common characteristics and risks. Furthermore, using of Yule Q coefficient enabled us to measure the correlation among the binary chronic disease data.

Our study does not come without its limitations. Although using the self-reported weight and measured height in the baseline information was economical and straightforward, it might underestimate the real value for BMI. Individuals mostly tend to report less weight, then the real value for BMI goes far from the reported one which might be subject to recall bias [30]. Moreover, since chronic disease prevalence was mainly based on self-reported data, disease severity was not considered. Furthermore, we only used the birth year of individuals. We did not have any information about childhood diet, mother's health or exposure to adversity, birth weight, separation of the child from the parent, and others, which may determine MM later in life. Since we examined the longitudinal association of birth phases with MM, we identified the temporal sequence between exposure and outcome, which might support a potential causal link. To preclude any cause and effect interpretation, other covariates were simultaneously used in model adjustment.

Moreover, although we used the standardized age variable in our models, we need to interpret our results cautiously since the prevalence and patterns of MM are influenced by age.

## Conclusion

This research offers insight into differences in the MM prevalence for individuals aged 65–71 years born in different periods before, during, and after World War II. Adverse circumstances experienced during the late war period may have contributed to the higher MM prevalence in adult life. Moreover, our findings suggest three main disease clusters: i. Joint and psychosomatic diseases (joint, neurological, anxiety, depression); ii. Cardiometabolic diseases (heart, stroke, diabetes, hypertension); iii. Other internal organ diseases (lung, gastrointestinal, kidney, and liver). Although the adverse situation of World War II and famine may increase MM risk at retirement age, more comprehensive childhood and life course circumstances are required to explain long-term health consequences. Future research shall investigate potential interactions between risk factor profiles during adult life and adverse early life exposures on MM. Furthermore, our results on the diseases clustering focusing on three significant clusters of MM call for further investigation, specifically their association with genetic effects, environmental factors, and polypharmacy.

## Supplementary Information

**Additional file 1. **

## Data Availability

Data is not openly available, and participants' data privacy is protected by data-protection standards established by the ethics committee of Bavarian Chamber of Physicians, Munich. However, data could be available upon request through a project agreement with KORA (http://epi.helmholtz-muenchen.de/koragen/) and the KORA Board approval.

## Abbreviations

MM: Multimorbidity
BMI: Body mass index
KORA: Cooperative Health Research in the Region of Augsburg
