# Supplementary material for: Impact of prenatal and childhood adversity effects around World War II on multimorbidity: results from the KORA-Age study
Source: BMC Geriatr. 2022 Feb 11;22:115. doi: 10.1186/s12877-022-02793-2 (PMC8832818; doi:10.1186/s12877-022-02793-2)
Supplement: Supplementary file 1 — Additional file 1. [file 12877_2022_2793_MOESM1_ESM.docx]

**Impact of prenatal and childhood adversity effects around World War II on multimorbidity: Results from the KORA-Age study**

Ava Arshadipour^1,2*^, BarbaraThorand^1,6^, Birgit Linkohr^1^, Susanne Rospleszcz^1,2^, Karl-Heinz Ladwig^1,3^, Margit Heier^1,4^, Annette Peters^1,2,5,6^

1. Institute of Epidemiology, Helmholtz Munich, German Research Center for Environmental Health, Neuherberg, Germany.
2. Institute for Medical Information Processing Biometry and Epidemiology (IBE), Ludwig-Maximilians-Universität München, Munich, Germany.
3. Department for Psychosomatic Medicine and Psychotherapy, Klinikum Rechts der Isar, Technical University of Munich, Munich, Germany.
4. KORA Study Centre, University Hospital of Augsburg, Augsburg, Germany.
5. German Center for Cardiovascular Disease Research (DZHK), Munich Heart Alliance, Munich, Germany.
6. German Center for Diabetes Research (DZD), München-Neuherberg, Germany.

**Journal: BMC Geriatrics**

§ Corresponding author

Ava Arshadipour,

1. Institute for Medical Information Processing Biometry and Epidemiology (IBE), Ludwig-Maximilians-Universität München, Munich, Germany.

Institute of Epidemiology, Helmholtz Munich, German Research Center for Environmental Health, Ingolstädter Landstraße 1, 85764 Munich, Germany

Tel.: +49 89 3187-49281, +49 1777369163

Email: a.arshadipour@campus.lmu.de, a.arshadipour2020@gmail.com

# Figure S1. Predicted probability of MM stratified by birth phases and sex

#
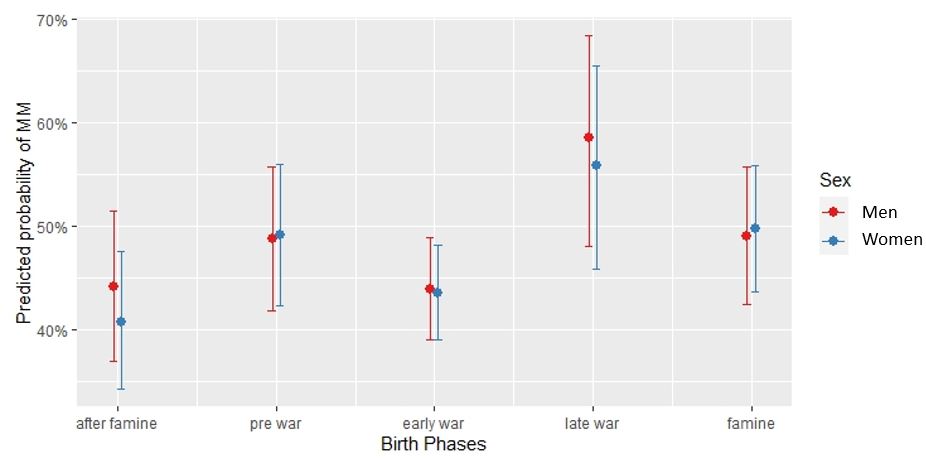


# The interaction of sex and birth phases for predicted probability of MM based on multiple logistic regression adjusted for age, sex, education, alcohol use, physical activity, BMI, smoking behavior and cognitive status. There were no significant differences (p < 0.05) in all levels of birth phases between men and women.

**Figure S2. Prevalence of multimorbidity and single chronic diseases stratified by sex for all participants (n=3,377) from KORA-Age 1 and KORA-Age 3.**

**Figure S3. Dendrogram based on the hierarchical clustering of chronic diseases using the average linkage method and Yule-Q coefficient for men.**


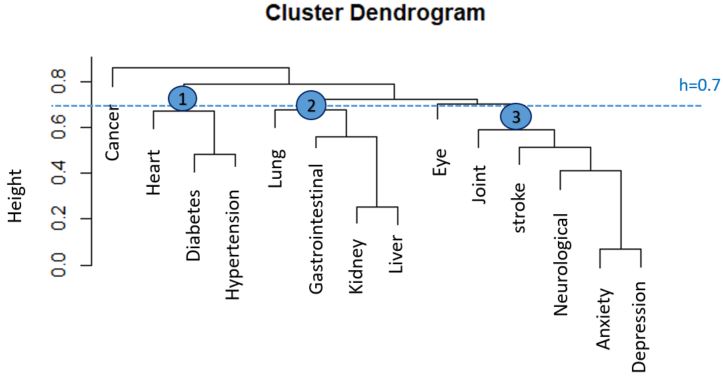


# The threshold (dotted line. h=0.7) was used to specify three main clusters.

**Figure S4. Dendrogram based on the hierarchical clustering of chronic diseases using the average linkage method and Yule-Q coefficient for women.**

**
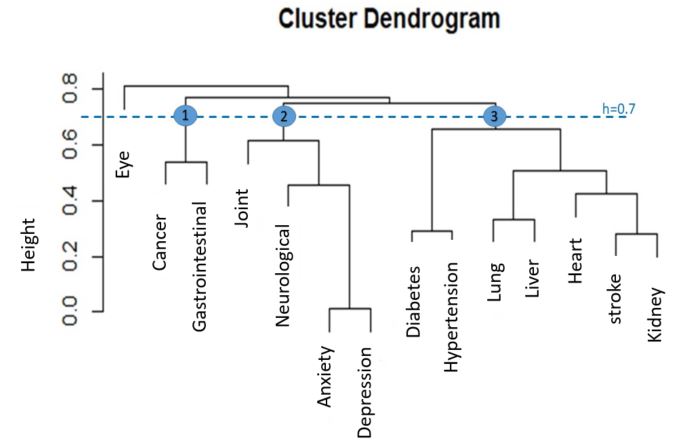
**

# The threshold (dotted line. h=0.7) was used to specify three main clusters.

**Figure S5. Dendrogram based on the hierarchical clustering of chronic diseases without hypertension for all participants using average linkage method and Yule-Q coefficient (Sensitivity analysis).**

**
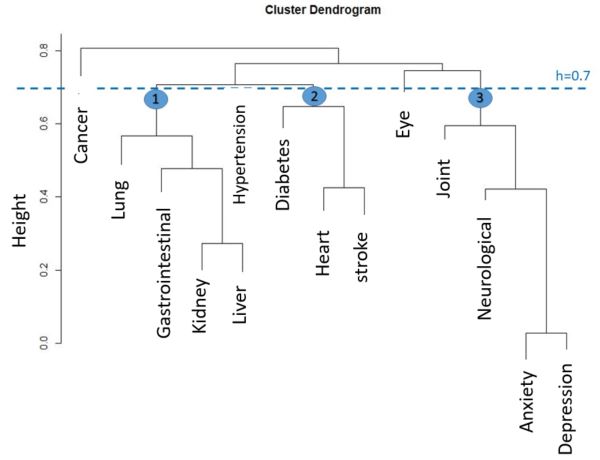
**

# The threshold (dotted line. h=0.7) was used to specify three main clusters.

**Figure S6. Dendrogram based on the hierarchical clustering of chronic diseases for all participants using the single linkage method and Yule-Q coefficient (sensitivity analysis).**

**
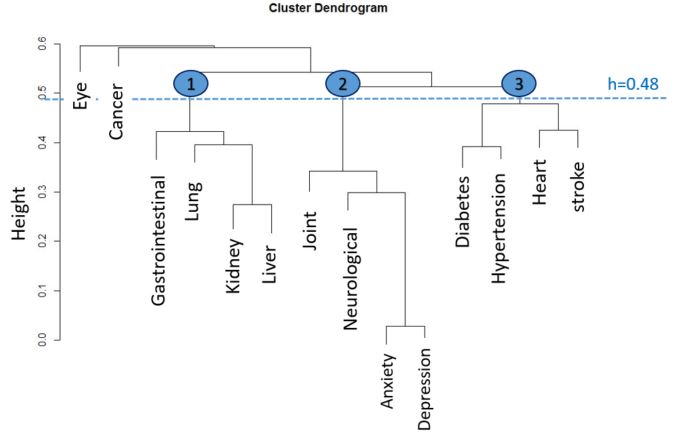
**

# The threshold (dotted line. h=0.7) was used to specify three main clusters.

**Figure S7. Dendrogram based on the hierarchical clustering of chronic diseases for all participants using Ward.d linkage method and Yule-Q coefficient (sensitivity analysis).
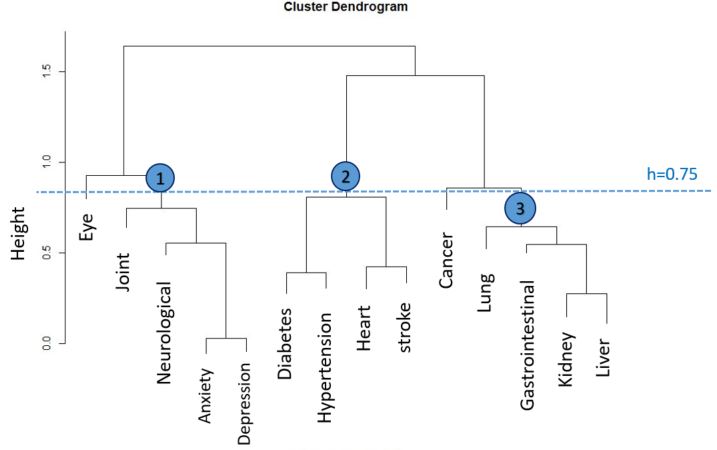
**

# The threshold (dotted line. h=0.7) was used to specify three main clusters.

**Table S1. The number of chronic diseases stratified by sex and birth phase variable for the whole data from KORA-Age 1 and Kora-Age 3.**

| Number of diseases | Total | | | Pre-war | | | Early war | | | Late war | | | Famine | | | After famine | | |
| --- | --- | --- | --- | --- | --- | --- | --- | --- | --- | --- | --- | --- | --- | --- | --- | --- | --- | --- |
|  | Total (3,377) | Male (1,616) | Female (1,761) | Total (684) | Male (327) | Female (357) | Total (1,236) | Male (608) | Female (628) | Total (283) | Male (136) | Female (147) | Total (574) | Male (267) | Female (307) | Total (600) | Male (278) | Female (322) |
| 0 | 638 (18.9%) | 312 (19.3%) | 326 (18.5%) | 112 (16.4%) | 56 (17.1%) | 56 (15.7%) | 243 (19.7%) | 128 (21.1%) | 115 (18.3%) | 42 (14.8%) | 21 (15.4%) | 21 (14.3%) | 97 (16.9%) | 38 (14.2%) | 59 (19.2%) | 144 (24%) | 69 (24.8%) | 75 (23.3%) |
| 1 | 1,072 (31.7%) | 516 (31.9%) | 556 (31.6%) | 202 (29.5%) | 99 (30.3%) | 103 (28.9%) | 432 (35%) | 207 (34%) | 225 (35.8%) | 65 (23%) | 30 (22.1%) | 35 (23.8%) | 167 (29.1%) | 90 (33.7%) | 77 (25.1%) | 206 (34.3%) | 90 (32.4%) | 116 (36.0%) |
| 2 | 873 (25.9%) | 404 (25.0%) | 469 (26.6%) | 186 (27.2%) | 84 (25.7%) | 102 (28.6%) | 311 (25.2%) | 149 (24.5%) | 162 (25.8%) | 85 (30%) | 41 (30.1%) | 44 (29.9%) | 151 (26.3%) | 71 (26.6%) | 80 (26.1%) | 140 (23.3%) | 59 (21.2%) | 81 (25.2%) |
| >=2 (Multi  morbidity) | 1,667 (49.4%) | 788 (48.8%) | 879 (49.9%) | 370 (54.1%) | 172 (52.6%) | 198 (55.5%) | 561 (45.4%) | 273 (44.9%) | 288 (45.9%) | 176 (62.2%) | 85 (62.5%) | 91 (61.9%) | 310 (54%) | 139 (52.1%) | 171 (55.7%) | 250 (41.7%) | 119 (42.8%) | 131 (40.7%) |
| >=3 | 794 (23.5%) | 384 (23.8%) | 410 (23.3%) | 184 (26.9%) | 88 (26.9%) | 96 (26.9%) | 250 (20.2%) | 124 (20.4%) | 126 (20.1%) | 91 (32.2%) | 44 (32.4%) | 47 (32%) | 159 (27.7%) | 68 (25.5%) | 91 (26.9%) | 110 (18.3%) | 60 (21.6%) | 50 (15.5%) |
| >=4 | 320 (9.5%) | 147 (9.1%) | 173 (9.8%) | 78 (11.4%) | 37 (11.3%) | 41 (11.5%) | 104 (8.4%) | 48 (7.9%) | 56 (8.9%) | 35 (12.4%) | 16 (11.8%) | 19 (12.9%) | 62 (10.8%) | 25 (9.4%) | 37 (12.1%) | 41 (6.8%) | 21 (7.6%) | 20 (6.2%) |
| >=5 | 124 (3.7%) | 57 (3.5%) | 67 (3.8%) | 30 (4.4%) | 12 (3.7%) | 18 (5%) | 44 (3.6%) | 22 (3.6%) | 22 (3.5%) | 17 (6%) | 9 (6.6%) | 8 (5.4%) | 21 (3.7%) | 10 (3.7%) | 11 (3.6%) | 12 (2%) | 4 (1.4%) | 8 (2.5%) |
| >=6 | 50 (1.5%) | 24 (1.5%) | 26 (1.5%) | 12 (1.8%) | 4 (1.2%) | 8 (2.2%) | 21 (1.7%) | 11 (1.8%) | 10 (1.6%) | 6 (2.1%) | 3 (2.2%) | 3 (2%) | 9 (1.6%) | 4 (1.5%) | 5 (1.6%) | 2 (0.3%) | 2 (0.7%) | 0 (0.0%) |

Phases were defined based on participant’s critical developmental age (prenatal gestation or the first two years of life) and the World War II situation in Germany.

**Table S2. Odds ratios and 95% confidence intervals for multimorbidity based on hierarchical logistic regression without hypertension for the whole participants (n=3,377) from KORA-Age 1 and KORA-Age 3. (Sensitivity analysis)**

| characteristics | | model 1 | model 2 | model 3 | model 4 |
| --- | --- | --- | --- | --- | --- |
| age | | 1.11 (0.95-1.15) |  | 1.10 (0.96-1.15) | 1.10 (0.96-1.15) |
| Birth phases (ref: After famine) | Pre-war |  | 1.64 (1.28-2.09) | 1.44 (0.96-2.16) | 1.64 (1.07-2.49) |
|  | Early war |  | 1.21 (0.96-1.52) | 1.17 (0.92-1.49) | 1.21 (0.94-1.56) |
|  | Late war |  | 2.3 (1.69-3.1) | 1.98 (1.22-3.19) | 2.14 (1.29-3.52) |
|  | Famine |  | 1.68 (1.30-2.17) | 1.56 (1.12-2.15) | 1.52 (1.08-2.14) |
| Female (ref: male) |  |  |  |  | 0.91 (0.75-1.08) |
| Education (ref: Low) | Middle |  |  |  | 0.99 (0.76-1.3) |
|  | High |  |  |  | 0.87 (0.65-1.18) |
| Alcohol consumption (ref: never or rare use) | Once a week |  |  |  | 0.99 (0.78-1.24) |
|  | Daily use |  |  |  | 0.66 (0.55-0.8) |
| Physical Activity (active) | Inactive |  |  |  | 1.44 (1.22-1.69) |
| BMI (ref: Underweight or normal ) | Overweight |  |  |  | 1.05 (0.87-1.27) |
|  | Obesity class I |  |  |  | 1.46 (1.16-1.84) |
|  | Obesity class II or III |  |  |  | 2.11 (1.52-2.91) |
| Smoking behavior(ref: never) | Active smoker |  |  |  | 1.28 (0.97-1.67) |
|  | Ex-smoker |  |  |  | 1.41 (1.19-1.68) |
| Cognitive status(ref: good) | Mildly impaired |  |  |  | 1.7 (1.27-2.27) |
|  | Impaired |  |  |  | 1.31 (0.80-2.17) |
| AIC | | 4,107.9 | 4,103.6 | 4,105 | 3,873.2 |

# Phases were defined based on participant’s critical developmental age (prenatal gestation or the first two years of life) and the World War II situation in Germany.

# Table S3. Resulting for multimorbidity risk based on hierarchical logistic regression (odds ratio and 95% confidence intervals) and generalized linear mixed models (GLMMs) for the whole participants (n=3,377) from KORA-Age 1 and KORA-Age 3 (Sensitivity analysis).

| Characteristics | | logistic regression model | Generalized linear mixed model with phase as random effect | Generalized linear mixed model with phase nested in study as random effect |
| --- | --- | --- | --- | --- |
|  |  |  |  |  |
| Standardized age | | 1.11 (0.96-1.27) | 1.26 (0.80,1.26) | 1.26 (1.19,1.33) |
| Birth phases (ref: After famine) | Pre-war | 1.35 (0.92-1.98) | Random effect | Random effect |
|  | Early war | 1.08 (0.87-1.34) |  |  |
|  | Late war | 1.83 (1.15-2.91) |  |  |
|  | Famine | 1.35 (0.99-1.84) |  |  |
| Female (ref: male) | | 0.98 (0.83-1.16) | 0.97 (0.72,1.14) | 0.99 (0.81,1.13) |
| Education (ref: low) | Middle | 0.82 (0.63-1.05) | 0.82 (0.62,1.05) | 0.83 (0.58,1.07) |
|  | High | 0.72 (0.55-0.95) | 0.75 (0.49,9.96) | 0.73 (0.48,1.02) |
| Alcohol consumption (ref: never or rare use) | Once a week | 0.99 (0.81-1.20) | 0.97 (0.80,1.11) | 0.95 (0.75, 1.18) |
|  | Daily use | 0.76 (0.64-0.91) | 0.74 (0.61,0.89) | 0.72 (0.57,0.91) |
| Physical Activity (active) | Inactive | 1.47 (1.26-1.72) | 1.53 (1.24,1.84) | 1.50 (1.38,1.68) |
| BMI (ref: underweight or normal) | Overweight | 1.32 (1.11-1.57) | 1.30 (1.08,1.52) | 1.28 (1.05,1.47) |
|  | Obesity class I | 1.92 (1.55-2.38) | 1.89 (1.47,2.28) | 1.75 (1.41,2.11) |
|  | Obesity class II or III | 3.09 (2.21-4.33) | 3.01 (2.04,4.15) | 3.14 (2.01,4.04) |
| Smoking behavior (ref: never smoker) | Active smoker | 1.34 (1.05-1.72) | 1.39 (1.11,1.34) | 1.39 (1.14,1.63) |
|  | Ex-smoker | 1.36 (1.16-1.59) | 1.34 (1.04,1.55) | 1.34 (1.19,1.50) |
| Cognitive status (ref: good) | Mildly impaired | 1.43 (1.07-1.92) | 1.39 (1.03,1.85) | 1.33 (1.01,1.78) |
|  | Impaired | 1.32 (0.81-2.18) | 1.32 (0.79,2.15) | 1.29 (0.77,2.07) |

# Phases were defined based on participant’s critical developmental age (prenatal gestation or the first two years of life) and the World War II situation in Germany.
